# A Highly Efficient Xylan-Utilization System in *Aspergillus niger* An76: A Functional-Proteomics Study

**DOI:** 10.3389/fmicb.2018.00430

**Published:** 2018-03-22

**Authors:** Weili Gong, Lin Dai, Huaiqiang Zhang, Lili Zhang, Lushan Wang

**Affiliations:** ^1^The State Key Laboratory of Microbial Technology, Shandong University, Jinan, China; ^2^State Key Laboratory of Biochemical Engineering, Institute of Process Engineering, Chinese Academy of Sciences, Beijing, China

**Keywords:** xylan-degrading isoenzyme, sugar transporter, transcription activator XlnR, *Aspergillus niger* An76, xylan

## Abstract

Xylan constituted with β-1,4-D-xylose linked backbone and diverse substituted side-chains is the most abundant hemicellulose component of biomass, which can be completely and rapidly degraded into fermentable sugars by *Aspergillus niger*. This is of great value for obtaining renewable biofuels and biochemicals. To clarify the underlying mechanisms associated with highly efficient xylan degradation, assimilation, and metabolism by *A. niger*, we utilized functional proteomics to analyze the secreted proteins, sugar transporters, and intracellular proteins of *A. niger* An76 grown on xylan-based substrates. Results demonstrated that the complete xylanolytic enzyme system required for xylan degradation and composed of diverse isozymes was secreted in a sequential order. Xylan-backbone-degrading enzymes were preferentially induced by xylose or other soluble sugars, which efficiently produced large amounts of xylooligosaccharides (XOS) and xylose; however, XOS was more efficient than xylose in triggering the expression of the key transcription activator XlnR, resulting in higher xylanase activity and shortening xylanase-production time. Moreover, the substituted XOS was responsible for improving the abundance of side-chain-degrading enzymes, specific transporters, and key reductases and dehydrogenases in the pentose catabolic pathway. Our findings indicated that industries might be able to improve the species and concentrations of xylan-degrading enzymes and shorten fermentation time by adding abundant intermediate products of natural xylan (XOS) to cultures of filamentous fungi.

## Introduction

Xylan with complex and heterogeneous structure is the second most abundant polysaccharide in biomass. The xylan backbone is linked with D-xylose by β-1,4 glucosidic bonds and is usually substituted by diverse groups, including L-arabinose, 4-*O*-methyl-glucouronic acid, or acetyl. L-arabinose substituents are capable of forming individual links to xylose [(1→ 2) or (1→ 3)] or through double substitution [(1→ 2) and (1→ 3)]. Certain L-arabinoses also join with other arabinoses or ferulic acid molecules by a (1→ 5) linkage, thereby joining xylan to lignin (Dodd and Cann, 2009). Therefore, complete conversion of xylan into fermentable sugars to produce renewable biofuels and biochemicals requires the action of complex xylan-degrading enzymes (Beg et al., 2001; Saha, 2003).

Due to the complex structure of xylan, diverse enzymes, including backbone-degrading enzymes and auxiliary enzymes found in the carbohydrate-active enzyme database (http://www.cazy.org/), are required to convert xylan into its constituent sugars. Endo-β-1,4-xylanases (EC 3.2.1.8) and β-xylosidases (EC 3.2.1.37) are required to degrade the xylan backbone, whereas α-arabinofuranosidases (EC 3.2.1.55), α-glucuronidases (EC 3.2.1.39), or acetylxylan esterases (EC 3.1.1.6) are auxiliary enzymes essential for catalyzing the complete degradation of complex branched xylans (Juturu and Wu, 2012).

Genomic data suggests that *Aspergillus niger* is a good producer of multifunctional xylanolytic enzymes (Baker, 2006). Specifically, the genomes of *A. niger* CBS 513.88 and *A. niger* An76 (Pel et al., 2007; Gong et al., 2016a) contain the full complement of enzymes, including numerous isozymes distributed among diverse families, required for complete xylan degradation. However, secretion of xylan-degradation enzymes requires large amounts of energy, making this process a risky investment for *A. niger* (Dekel and Alon, 2005); therefore, the recognition of heterogenous xylan and its rapid conversion into energy constitutes a natural selection process for *A. niger*. However, the molecular mechanisms associated with heterogenous xylan recognition and its efficient uptake and metabolism in *A. niger* remain to be elucidated.

Large-scale transcriptome studies demonstrated that a carbon-source-dependent response was conserved among *Aspergillus* spp. (Andersen et al., 2008). Simple sugars, such as glucose, xylose, and arabinose, have been utilized to study the global transcriptional response of *Aspergillus* spp. (Salazar et al., 2009; Battaglia et al., 2011). Xylose and arabinose were identified as good transcriptional inducers of endoxylanase and arabinan-degrading enzymes, respectively (Battaglia et al., 2011); however, the different responses of isozymes having different structures and product profiles have seldom been distinguished (Gong et al., 2016b). Additionally, the xylan-degradation process is dynamic, leading to rapid changes in extracellular hydrolysate species, especially 4-O-methyl-glucouronic acid substituted xylooligosaccharide (XOS) and unsubstituted XOS (xylobiose, xylotriose, xylotetraose, xylopentaose), which was proved with Fluorescence-assisted carbohydrate electrophoresis (FACE) and electrospray ionization mass spectrometry (Gong et al., 2016b). A previous study of *Neurospora crassa* reported that cellodextrins acted as inducers of cellulases (Znameroski et al., 2012), whereas few studies have focused on the effect of XOS on xylan utilization (Herold et al., 2013). Earlier studies also showed that the expression of transporters is modulated by cellulose, sophorose, and glucose and play important roles in cellulase induction (Gupta et al., 2016). The response and roles of transporters in xylan utilization deserve further study. Most transcriptome studies were performed at earlier specific growth stages while ignoring the entire growth process and complicated post-transcriptional modifications. Furthermore, intracellular processes require extended periods to allow the complete catabolism of imported sugars. Therefore, dynamic functional proteomics may be able to provide more comprehensive information to allow the study of factors affecting xylan utilization.

Here, xylan, xylan hydrolysates (xylose, arabinose, and substituted XOS), pentose metabolic intermediates (xylitol), and polymer derivatives [carboxymethylcellulose sodium (CMC)] were used as carbon sources to culture *A. niger* An76. Our acquisition and analysis of the secreted proteins, sugar transporters, and intracellular proteins provided new insights into the processes involved in the highly efficient utilization of heterogenous xylan in *A. niger* An76.

## Materials and methods

### Strains and cultivation conditions

Spores (8 × 10^7^/mL) of *A. niger* An76 (Wang and Gao, 1990) were kept in 20% (v/v) glycerol at −80°C. Liquid medium (1 L) containing NaNO_3_ (5.95 g), KCl (0.522 g), KH_2_PO_4_ (1.497 g), MgSO_4_·7H_2_O (0.493 g), yeast extract (5 g), casamino acids (2 g), 1 mL of trace-element solution, and 1% (w/v) of different carbon sources, including glycerol, glucose, arabinose, xylose, xylitol, XOS, xylan, cellobiose, and CMC or a series of concentrations for xylose (0.05, 0.15, 0.75, 1%), were used to culture *A. niger* An76 at 30°C as previously described (Wang and Gao, 1990). Spores (200 μL) were used to inoculate 200 mL liquid medium in triplicate (three biological replicates), with samples (600 mL) collected every 24 for 120 h to analyze the effects of carbon source or concentration on protein production by *A. niger* An76. Arabinose, xylose, xylitol, and xylan were obtained from Futaste (Shandong, China), XOS, which is a mixture of xylobiose, xylotriose, xylotetraose, xylopentaose and substituted components with electrophoretic mobility between xylose and xylobiose, xylotriose and xylotetraose as shown in Figure S1, was provided by LONGLIVE (Shandong, China). All other reagents were purchased from Songon (Shanghai, China).

To obtain mycelia used for RNA extraction, liquid medium (100 mL) with glycerol (1 g) as a carbon source was utilized to preculture spores (100 μL) at 30°C for 24 h. Mycelia obtained before induction were collected as a reference sample (0 h), followed by transfer of the mycelia to induction medium containing 1% xylose or 1% XOS as a carbon source. Samples were harvested following induction for 2, 4, and 6 h, and liquid nitrogen was employed to immediately freeze the mycelia. The samples were collected from three batches and every sample in each batch was collected in duplicate, all the harvested samples were biological replicates for qPCR analysis.

### Determination of various cultivation parameters and enzyme activities

Biomass was characterized using the dry weight of fungal mycelium (Broekaert et al., 1990). Gauze was used to filter the mycelium, and they were dried together at 50°C until a constant weight was obtained. The remaining culture supernatant was harvested to determine pH, concentrations of reducing sugars and proteins, xylanase and arabinofuranosidase activities, the various cultivation parameters of samples in each biological replicate were determined three times (three technical replicates). A pH meter (Rex Electric Chemical, Shanghai, China) was used to determine the pH of the centrifuged culture medium.

The concentrations of the reducing sugars and xylanase activity were measured using the DNS method as previously described (Gong et al., 2015). The standard curve was prepared using a xylose standard, and 1% (w/v) beechwood xylan with (4-O-methyl)glucuronic acid as substituents (Sigma–Aldrich, St. Louis, MO, USA) in citric acid/disodium hydrogen phosphate (pH 5.0) was used as the substrate to determine xylanase activity (the reaction mixture including 400 μL extracellular protein solution [0.2 mg/mL] and 600 μL substrate was incubated at 50°C for 30 min). The reaction was terminated by submerging in boiling water for 10 min. The international unit (IU) was employed to define enzyme activity, one IU of xylanase activity was equal to 1 μM xylose released from xylan substrate by xylanase in 1 min, at optimum temperature (50°C) and pH (5.0). Protein concentration was determined by the Bradford method (Gong et al., 2015), and bovine serum albumin was used as standard substance to obtain a standard curve.

To measure arabinofuranosidase activity, *p*-nitrophenol-α-L-arabinofuranoside (*p*NPA, 2 mm/mL; Sigma–Aldrich, St. Louis, MO, USA) in citric acid/disodium hydrogen phosphate (pH 5.0) buffer was used as a substrate, with *p*NP (Sigma–Aldrich) used to prepare a standard curve. The reaction system included 50 μL *p*NPA, 50 μL (0.2 mg/mL) protein solution, and 100 μL buffer solution (pH 5.0) and was incubated at 50°C for 30 min. The reaction was terminated by addition of Na_2_CO_3_ (10% [w/v]), and products were detected at 420 nm using a microplate reader (Tecan Schweiz AG, Männedorf, Switzerland).

Statistical tests for dry weight, protein concentrations/enzyme assays, reducing sugar analysis were performed with one-way ANOVA method in GraphPad prism 5.0.

### Determination of reducing-sugar species with fluorescence-assisted carbohydrate electrophoresis (FACE)

Changes in reducing-sugar species in the XOS culture supernatant were detected by FACE (Zhang and Wang, 2015). Supernatant (5 μL) was fluorescently labeled in the dark for 1 h with 7-amino-1,3-naphthalenedisulfonic acid monopotassium salt monohydrate (5 μL, 0.2 M) dissolved in 15% acetic acid, then NaCNBH_3_ solution (1 M, 5 μL) in dimethyl sulfoxide was incubated with the mixture at 40°C for 12 h, the labeled products (6 μl/well) were subjected to electrophoresis with a miniaturized vertical gel system utilizing amini-PROTEAN 3 PowerPac Basic Power Supply (Bio-Rad, Hercules, CA, USA), the polyacrylamide gels were made as described in (19). 50% w/v sucrose was used as the loading buffer, and the running buffer was composed of 25 mM Tris-HCl (pH 8.3) and 192 mM glycine, the gels were scanned with a ChemiDoc™ MP imaging system (Bio-Rad) and stored in TIF format (Gong et al., 2016b).

### Detection of xylanase isozymes by native polyacrylamide gel electrophoresis (PAGE)

Detection of changes in xylanase isozymes secreted by *A. niger* An76 on different carbon sources was determined by native PAGE as previously reported. Xylan (1% [*w/v*]; Futaste) in citric acid/disodium hydrogen phosphate (pH 5.0) was utilized as the substrate, and the loading quantity of extracellular protein solution (0.2 mg/mL) was 10 μL/well. After electrophoresis, the gels were immersed in substrate (60 mL) at 50°C for 30 min. The methods used for gel staining, destaining, and scanning were described previously (Gong et al., 2015).

### Extraction of the secreted proteins and the whole-cell proteomics

The culture medium (600 mL) for each carbon source or different xylose concentrations at 120 h was filtered with eight layers of gauze, and the filtered liquid was collected for extracellular-protein analysis. The hyphae were grinded with liquid nitrogen, and the powder was dissolved in acetone (Dingguo, Beijing, China) supplemented with 13.3% (w/v) trichloroacetic acid (Sigma-Aldrich) and 0.093% (v/v) β-mercaptoethanol (Dingguo) overnight. The dissolved hypha was centrifuged at 14,000 rpm for 20 min at 4°C, the supernatant was discarded, and the pellet washed twice with pre-cooled acetone supplemented with 0.07% (v/v) β-mercaptoethanol. The pellet was allowed to dry at room temperature for 5 min prior to dissolving for 1 h in 30 mL lysis buffer containing 7 M urea, 2 M thiourea, 4% (w/v) CHAPS (Dingguo), 0.8% ampholytes, 20 mM Tris, and 20 mM dithiothreitol (Sigma-Aldrich), followed by ultrasonication for 15 min. The samples were then centrifuged at 20,000 g for 30 min at 16°C, and the supernatant was collected for sugar transporters and intracellular proteins analysis.

### Analysis of proteins by liquid chromatography tandem mass spectrometry (LC-MS/MS)

The acquired secreted proteins, sugar transporters and intracellular proteins were concentrated with 3-kDa cutoff ultrafiltration tubes (Millipore, Eschborn, Germany), and precipitated with trichloroacetic acid, then the obtained protein powder was dissolved in ultrapure water with protein concentration <10 μg/μL, 10 μL dissolved protein solution was taken to be mixed with 50 μL degeneration buffer, the degeneration buffer consisted of 0.5 M Tris–HCl, 2.75 mM EDTA, 6 M guanidine–HCl, then the proteins were reduced by adding 30 μL of 1 M dithiothreitol at 37°C for 2 h, and 50 μL iodoacetamide (1 M) was utilized to alkylate the samples in the dark for 1 h, the treated proteins were washed with 360 μL of NH_4_HCO_3_ (25 mM) on a Microcon YM-10 membrane (3-kDa cutoff) at 1,000 × *g* for 15 min, trypsin dissolved in 50 mM glacial acetic acid (0.5 μg/μL) was used to digest the washed proteins with a ratio of 1:25 (*w*/*w*) at 37°C overnight, the peptides were desalted with 50% (*v/v*) acetonitrile (ACN) and 0.1% (*v/v*) trifluoroacetic acid through a C_18_ Ziptip, after desalination, the peptide samples were dissolved in 10 μL of 0.1% (*v/v*) trifluoroacetic acid (Gong et al., 2015).

A Prominence nano LC system (Shimadzu, Tokyo, Japan), equipped with a custom-made silica column (75 μm × 15 cm) filled with Reprosil-Pur 120 C18-AQ (particle size 3 μm; Dr. Maish, Germany), was used to separate peptides, mobile phases were solvent A (2.0% ACN in water [*v/v*] with 0.1% [*v/v*] formic acid) and solvent B (98% ACN in water [*v/v*] with 0.1% [*v/v*] formic acid), the procedure of stepping gradient elution was set as: 2% (*v/v*) solvent B (0.0–5.0 min), 2–15% (*v/v*) solvent B (5.0–25.0 min), 15–40% solvent B (25.0–55.0 min), 40–98% (*v/v*) solvent B (55.0–60.0 min), 98% solvent B (60.0–70.0 min), 98–2% (*v/v*) solvent B (70.0–75.0 min), and 2% (*v/v*) solvent B (75.0–90.0 min) at a flow of 300 nL/min. All separated peptides were sprayed into the LTQ-Orbitrap Velos Pro ETD mass spectrometer (Thermo Scientific, MA, Germany) via a nanospray ion source with electrospray voltage of 2 kV and transfer capillary temperature of 275°C. The MS worked in data-dependent acquisition mode with Xcalibur 2.2.0 software (Thermo Scientific). Fullscan MS spectra (from 400 to 1800 m/z) were detected in the Orbitrap with a resolution of 60,000 at 400 m/z. The 10 most intense precursor ions greater than the threshold of 5,000 counts in the linear ion trap were selected for MS/MS fragmentation analysis at a normalized collision energy of 35%, and charge state screening parameters of +2 to +7 selected for MS/MS was applied. In order to avoid repetitively selecting peptides, dynamic exclusion was used within 60 s. A total of three biological replicates and two technical replicates were performed for each sample.

### Database search

Database searches used the software of Proteome Discoverer version 1.4 (Thermo Fisher Scientific, Waltham, MA, USA) with the Sequest HT as search engine. The reference database with 10,496 proteins was predicted from the genome of *A. niger* An76 sequenced by our lab and deposited in the DNA Data Bank of Japan (Gong et al., 2015, 2016a). The MS/MS search was conducted in accordance with the following settings: (i) trypsin was used to digest the proteins allowing two missed cleavages, (ii) a precursor mass tolerance of 10 ppm and a fragment mass tolerance of 0.8 Da were set for mass tolerance, (iii) oxidation of methionine was chosen as the dynamic modification and carbamidomethyl of cysteine residues was selected as the fixed modification. Only peptides with at least six amino acid residues showing 95% certainty (*q* ≤ 0.05) were included in the results, the Percolator in Sequest HT was assigned to obtain Peptide Probabilities (Käll et al., 2007). At least one peptides (*q* < 0.05) was needed to be considered for protein identification and the false discovery rate (FDR) was set as 1%, the Protein Prophet algorithm was utilized to calculate Protein probabilities (Nesvizhskii et al., 2003).

Peptide spectrum matches (PSMs), the resulting matches between experimental and theoretical spectra, was used for quantitation because the likelihood of spectral count is a good measure of relative protein abundance as reported in previous studies (Liu et al., 2004; Ivanov et al., 2014; Madsen et al., 2015), therefore, the number of PSMs per protein is proportional to protein abundance, but the presence of shared peptides or peptide sequences common to multiple proteins in the database used to interpret MS/MS spectra leads to inaccurate inference of protein abundance, this problem was solved by protein groups that involve shared peptides along with an Occam's razor constraint, and the solution method was embodied in the software ProteinProphet as reported by Nesvizhskii (Nesvizhskii et al., 2003). Significant difference (*P* < 0.05) was calculated by statistical analysis using *t*-test and one-way ANOVA methods in GraphPad prism 5.0 (Song et al., 2016).

### RNA extraction and cDNA synthesis

Collected mycelia were used to extract RNA by the Trizol method (Rio et al., 2010). All reagents, including trizol, chloroform, isopropyl alcohol, ethanol, and RNAase-free water, were purchased from Dingguo. RNA concentration was determined using a NanoDrop 1000 (Thermo Fisher Scientific).

cDNA was synthesized using the PrimeScript RT reagent kit with gDNA Eraser (Takara, Dalian, China) using a template-RNA concentration of 50 ng/μL.

### Quantitative polymerase chain reaction (qPCR) analysis

LightCycler 480 (Roche, Basel, Switzerland) was used to perform qPCR analysis. Primers employed in this study are listed in Table S1. The amplification mixture (25 μL) contained 12.5 μL SYBR Premix ExTaq II (Takara), 1 μL forward primer (10 μM), 1 μL reverse primer (10 μM), 2 μL cDNA template (50 ng/μL), and 8.5 μL sterilized distilled water. The cycling reaction involved a 2-min initial denaturation at 95°C, followed by 40 cycles at 94°C for 30 s, 55°C (g219.t1, g1617.t1, and g3669.t1) or 60°C (g3399.t1 and g3648.t1) for 30 s, and 72°C for 30 s. All samples were processed in three technical replicates. Relative transcription analysis was calculated with the equation:

$$\begin{array}{l} 2^{-[\text{induction}\Delta \text{Ct}(\text{target gene}-\text{reference gene})-\text{control}\Delta \text{Ct}(\text{target gene}-\text{reference gene})]}. \end{array}$$

Target genes included the transcription activator (*xlnR*; g3648.t1), D-xylose reductase (*xyrA*; g219.t1), L-arabitol dehydrogenase (*ladA*; g1617.t1), sorbitol/xylitol dehydrogenase (*xdhA*; g3399.t1), and sorbitol/xylulose reductase (*lxrC*; g3669.t1). Glyceraldehyde-3-phosphate dehydrogenase (*gapdh*; g7576.t1) was utilized as a reference gene. Error bars indicated the standard deviations. Serial dilutions of cDNA samples (10^−1^-10^−7^) were performed to determine amplification efficiency for each primer pairs, the amplification efficiency was guaranteed in the range from 95 to 105% *t*-test method was utilized to calculate the significant difference (*P* < 0.05) of gene expression in qRT-PCR analysis.

## Results

### Determination of various cultivation parameters for *A. niger* An76 grown on different carbon sources

**Figure 1:**
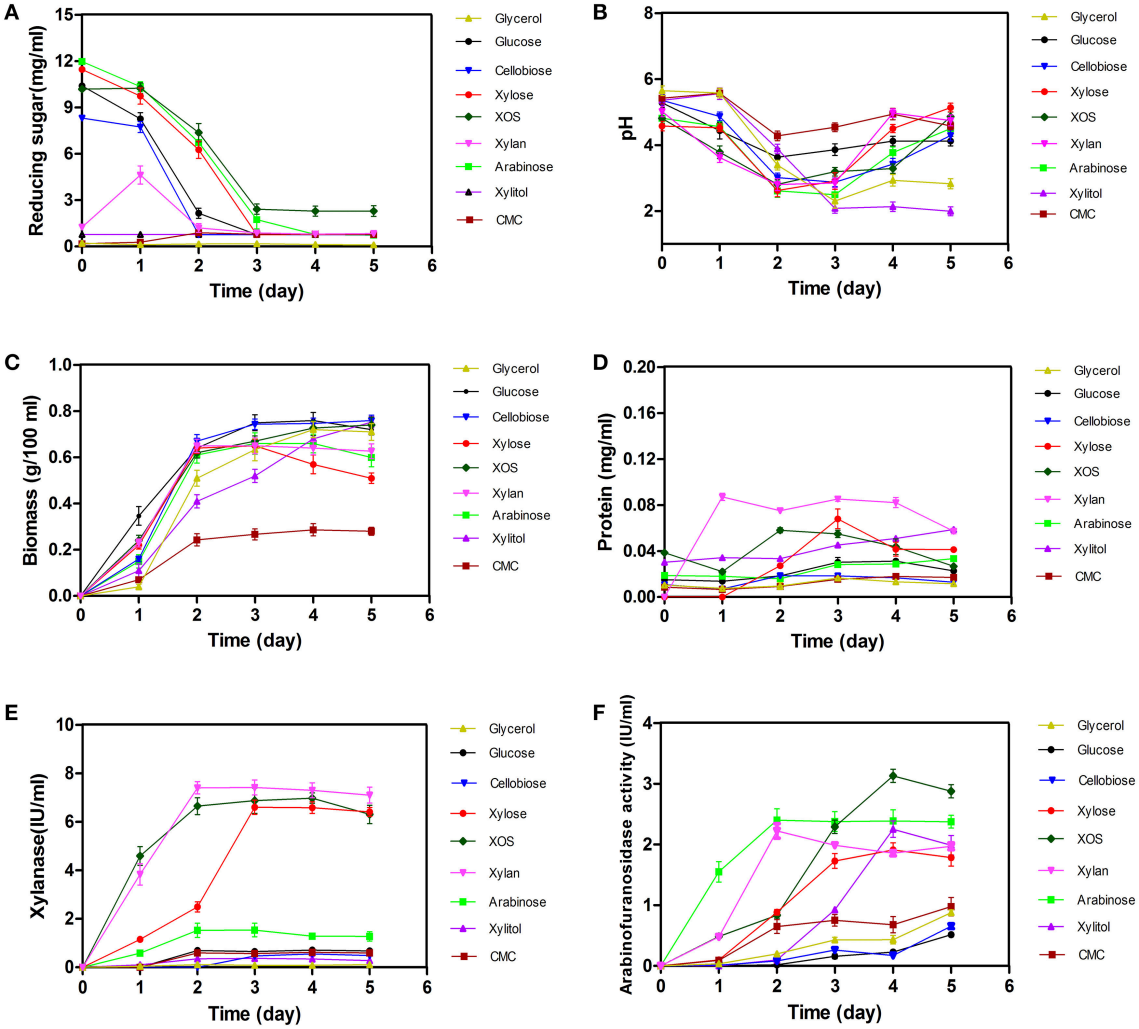
Time-dependent determination of changes in biomass, pH, concentrations of extracellular reducing sugars and proteins, and the enzyme activity of xylanases and arabinofuranosidases in *Aspergillus niger* An76 grown on nine different carbon sources. **(A)** Reducing sugars, **(B)** pH, **(C)** Biomass, **(D)** Proteins, **(E)** Xylanases, and **(F)** Arabinofuranosidases.

Changes in biomass, extracellular pH, reducing sugars, proteins, xylanase activity, and arabinofuranosidase activity associated with *A. niger* An76 were shown in Figure 1.

As shown in Figure 1A, the concentration of monosaccharide and disaccharide decreased sharply on day 2 (*P* = 0.0189), and the reducing ends of polysaccharide CMC increased in initial 2 days, then decreased in later stage, suggesting that these carbon substrates could be utilized by *A. niger* An76. Additionally, glucose, xylose, and arabinose were almost completely depleted on days 3–5, respectively. The discrepancy in utilization efficiency was consistent with that observed in most microbes (Li et al., 2014).

Along with the consumption of carbon sources, we observed a decrease in pH during the initial 3 days, followed by a slight increase over the final 2 days (Figure 1B). Notably, on day 3, the pH decreased by ~2 to ~3 in cultures on each of the substrates tested. The drop in pH may be related to the production of citric acid, similar to that observed in *A. niger* ATCC1015 (Andersen et al., 2011).

Determination of time-dependent changes in biomass revealed an increase on day 2 (*P* = 0.6845, Figure 1C), indicating that the assimilated sugars were rapidly metabolized to support growth. In addition to growth, low concentrations of proteins were secreted, whereas protein concentrations released following utilization of glycerol, glucose, cellobiose, or CMC were much lower than those observed in the presence of other carbon sources (*P* < 0.01, Figure 1D). The different protein-inducing abilities associated with each carbon source implied the expression-level discrepancy of enzymes among the secreted proteins.

Compared with the different protein-inducing abilities associated with each carbon source, we observed a greater discrepancy in xylanase activity (*P* < 0.01, Figure 1E). Xylanase activities induced by the presence of xylose, XOS, and xylan were higher than those induced by other carbon substrates. Enzyme activity induced by xylose was detected on day 1, followed by a steady increase in activity until day 5, similar to previous reports (Xing et al., 2013); however, xylanase activity induced by XOS and xylan on day 1 was ~4-fold higher than that induced by xylose, and the xylanase activity induced by XOS and xylan on day 2 reached the level induced by xylose on day 3, indicating the advantage of using XOS to shorten xylanase-expression time.

Arabinofuranosidase (Figure 1F) exhibited enzyme activity on day 1 on arabinose, which was much higher than that on the other carbon sources, and continued to increase gradually up to day 5. Although arabinose, xylose, xylitol, XOS, and xylan exhibited adequate induction of arabinofuranosidase, that induced by XOS and arabinose exceeded that of the other carbon sources (*P* < 0.01).

### Analysis of the effects of carbon sources on xylanase production by zymography using native page

Endoxylanase isozymes were detected by native PAGE (Figure S2), and XynC, XynB, and XynA were in accordance with Xyn1 (GH10), Xyn2 (GH11) and Xyn3 (GH11), respectively, reported in previous studies (Xing et al., 2013; Gong et al., 2016b). In previous study (Xing et al., 2013), the genome of *A. niger* An-76 was not sequenced, the three xylanases secreted by the *A. niger* An76 were named by Xyn1, Xyn2, Xyn3. In 2016, the genome of *A. niger* An-76 was sequenced and deposited into NCBI (Gong et al., 2016a). We refer to a series of references and find that the standard 3-letter enzyme names start with a capital and are followed by a capital (e.g., A, B) (Battaglia et al., 2011), therefore, Xyn1, Xyn2, and Xyn3 were renamed after XynC, XynB, and XynA, respectively (Figure S3). As previously reported, xylanase production is time- and carbon-source-dependent (Xing et al., 2013; Gong et al., 2015). Glycerol, glucose, and cellobiose minimally induced the formation of xylanases and resulted in only trace amounts of XynB (GH11) (Figures S2A–C), whereas xylose, XOS, and xylan more easily induced the production of high concentrations of xylanases (Figures S2D–F). However, the main side chain of xylan (arabinose) and the key metabolite of pentose (xylitol) only induced low concentrations of XynB (Figures S2G,H), whereas CMC, containing similar carboxylic functional groups as xylan, was able to continuously induce the formation of XynC and XynB (Figure S2I).

The three xylanase isozymes displayed different characteristics. XynB (GH11) was induced by all carbon sources, except glycerol, indicating that XynB plays a primary role in degrading xylan and identifying numerous enzymes homologous to XynB, which is consistent with previous reports (Krisana et al., 2005; Fu et al., 2012; Takahashi et al., 2013). Although XynA is also in the GH11 family, its expression differed from that of XynB, corresponding to the different product profiles of XynB and XynA reported previously (Gong et al., 2016b). XynA activity was dramatically enhanced by xylose, XOS, and xylan, but reduced by substrates derived from cellulose, such as glucose, cellobiose, and CMC. These results were in agreement with those from previous studies showing that XynA was induced by 1% xylose and 0.05% cellobiose after 48 h, while up to 72 h in the presence of 1% xylose and 1% cellobiose (Xing et al., 2013), suggesting that XynA was more sensitive to glucose-mediated carbon-catabolite repression.

Additionally, we observed more flexible changes to XynC (GH10). On day 1, when grown on xylose, XynC expression levels decreased to below those observed in the presence of XOS, xylan, and CMC. The absence of XynC expression on days 3 and 4 might be related to the decrease in pH. In *Aspergillus* spp., production of certain hydrolytic enzymes is controlled by external pH (Mingot et al., 2001), and the formation of proteases can be triggered by ambient pH (van den Hombergh et al., 1997).

### LC-MS/MS analysis of extracellular xylanases induced by different concentrations of xylose and by XOS

**Figure 2:**
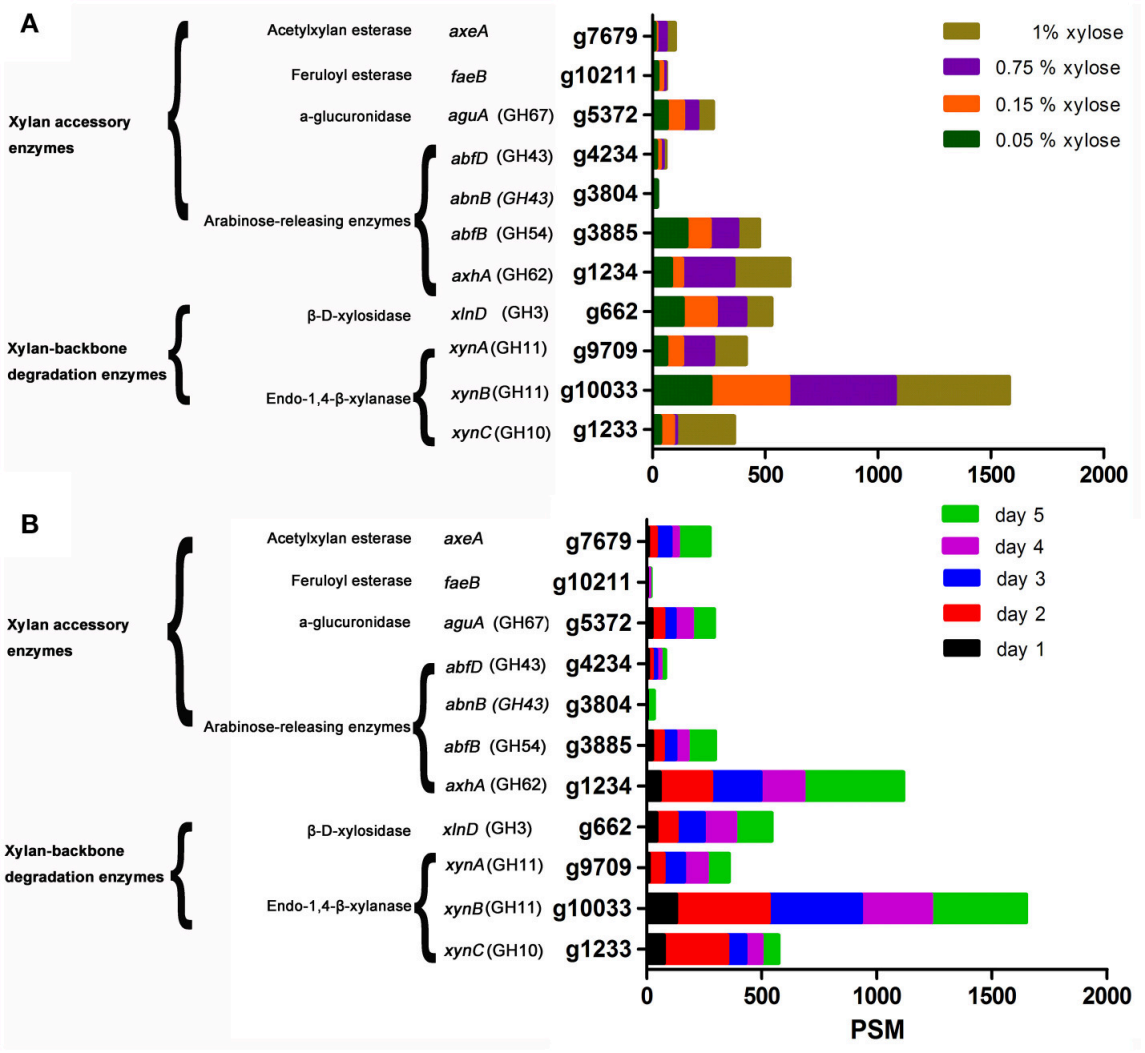
LC-MS/MS detection of the expression of xylan-degrading enzymes secreted by *Aspergillus niger* An76 on day 5 and grown on different xylose concentrations **(A)** Time-course analysis of the abundance of xylan-degrading enzymes produced by *A. niger* An76 on 1% XOS **(B)**.

Xylanase activity, as well as the expression levels of XynA, XynB, and XynC induced by XOS and xylan, was clearly higher than that induced by xylose on day 1 (Figure 1E and Figures 2D–F). To determine whether the advantage of XOS and xylan resulted from the continual release of low concentrations of xylose, the effects of xylose concentration on xylanase production were further investigated by characterizing the zymogram on day 2 (Figure S4) and the secretome on day 5 with LC-MS/MS. Additionally, enzymes were classified according to function, and their expression levels were characterized according to PSMs (Figure 2A, Table S2; Saykhedkar et al., 2012). Correlation analysis indicated that the relative xylanase activity on day 2 and the expression levels of XynA and XynB increased linearly with xylose concentration (Figures S4, S5), agreeing with previous zymography results (Xing et al., 2013). A previous transcriptional study of hydrolase-expression profiles in *A. niger* by Mach-Aigner et al. (Mach-Aigner et al., 2012) also reported that high xylose concentrations were favorable for the transcription of certain groups of genes. Therefore, on day 1, the higher expression level of xylanases induced by XOS was predicted to be not a consequence of the lower xylose concentration released by XOS, but it might have been induced by the imported XOS. Furthermore, in regard to the side-chain-degrading enzymes, the expression levels of AxhA (g1234.t1), AbfB (g38885.t1), and AguA (g5372.t1) were relatively high, but only increases in that of AxhA (GH62) were positively correlated with xylose concentration.

All extracellular xylan-degrading enzymes induced by XOS were analyzed over a time-course by LC-MS/MS (Figure 2B, Table S3). On day 1, we detected low concentrations of endoxylanases, whereas on day 2, the expression level increased by 2- to 4-fold and remained stable until day 5. We also observed gradual increases in arabinofuranosidase concentration, and compared with the total expression level of arabinofuranosidases (*axhA*, g1234.t1; *abfB*, g3885.t1; *abnB*, g3804.t1; *abfD*, g4234.t1) on day 1 (PSM = 84), the concentration of endoxylanase was much higher (PSM = 209; Figure 2B). Additionally, obvious endoxylanase activity was detected on day 1 relative to that determined for arabinofuranosidase activity, which was not observed until day 3 (Figures 1E,F). Moreover, the expression of glucuronidase (AguA; g5372.t1), acetylxylan esterase (AxeA; g7679.t1), and feruloyl esterase (FaeB; g10211.t1) was detected from day 2. These results showed that backbone-degradation enzymes played essential roles in the initial degradation stage, followed by the induction of side-chain-releasing enzymes. Furthermore, on day 5, we observed a significant increase in the XOS-induced expression of side-chain-releasing enzymes, including AxhA (g1234.t1), AbfB (g3885.t1), AbnB (g3804.t1), AguA (g5372.t1), and AxeA (g7679.t1), indicating that the expression of side-chain-cleaving enzymes may be closely related to the assimilation of substituted XOS released by backbone enzymes.

### Extracellular xylan-degrading enzymes induced by different carbon sources and detected by LC-MS/MS

Time-dependent analysis of the extracellular xylan-degrading enzymes induced by XOS revealed that the enzyme species and concentrations were highest on day 5. Therefore, to analyze the substrate specificity of the different enzymes, xylan-degrading enzymes induced by control substrates (glycerol, glucose, and cellobiose), a series of xylan constituents (xylan, XOS, xylose, and arabinose), intermediate metabolites of pentose (xylitol), and polymers with similar carboxylic acid substituents of xylan (CMC) on day 5 were grouped and compared (Table 1, Table S4). Based on previous reports (Saykhedkar et al., 2012; Gong et al., 2015), we classified the quantity of enzyme production into four levels: basal expression (PSM < 30), weak induction (30 < PSM < 100), medium induction (100 < PSM < 200), and strong induction (PSM > 200).

**Table 1:** The expression of xylan-degrading enzymes secreted by *Aspergillus niger* An76 on day 5 of growth on different carbon sources.

	**Activity**	**Gene name**	**Family**	**Genome ID**	**PSM** ± **sd**								
					**Glycerol**	**Glucose**	**Cellobiose**	**Xylose**	**XOS**	**Xylan**	**Arabinose**	**Xylitol**	**CMC**
**XYLAN-BACKBONE DEGRADATION ENZYMES**													
Xylanase	Endo-1,4-beta-xylanase	*xynC*	GH10	g1233.t1	4 ± 2.0	20 ± 5.1	15 ± 6.4	86 ± 15	73 ± 16	78 ± 15	0 ± 0.0	21 ± 3.4	87 ± 9.0
		*xynB*	GH11	g10033.t1	4 ± 2.0	30 ± 5.0	21 ± 6.0	301 ± 29	409 ± 30	360 ± 40	36 ± 7.0	69 ± 10	146 ± 15
		*xynA*	GH11	g9709.t1	0 ± 0.0	2 ± 0.0	0 ± 0.0	73 ± 19	84 ± 24	139 ± 16	0 ± 0.0	84 ± 4.0	20 ± 4.0
	β-xylosidase	*xlnD*	GH3	g662.t1	21 ± 5.7	1 ± 0.0	9 ± 2.7	153 ± 22	151 ± 22	543 ± 32	98 ± 12	61 ± 11	42 ± 7.0
		*xlnB*	GH43	g4894.t1	0 ± 0.0	7 ± 3.3	23 ± 3.5	9 ± 2.8	3 ± 0.98	4 ± 2.2	1 ± 0.63	0 ± 0.0	12 ± 3.1
**XYLAN ACCESSORY ENZYMES**													
	Arabinan endo-1,5-α-L-arabinosidase	*abnA*	GH43	g8588.t1	2 ± 0.4	0 ± 0.0	0 ± 0.0	0 ± 0.0	0 ± 0.0	0 ± 0.0	4 ± 1.4	0 ± 0.0	0 ± 0.4
	Endo-arabinase	*abnB*	GH43	g3804.t1	4 ± 1.4	128 ± 10	106 ± 7.0	90 ± 8.0	29 ± 8.0	6 ± 1.3	11 ± 2.8	0 ± 0.0	87 ± 4.3
	Arabinoxylan arabinofuranohydrolase	*axhA*	GH62	g1234.t1	27 ± 7.5	124 ± 11	76 ± 12	161 ± 26	432 ± 32	156 ± 11	169 ± 11	73 ± 6.0	76 ± 9.3
	a-L-arabinofuranosidase	*abfA*	GH51	g10075.t1	0 ± 0.0	0 ± 0.0	0 ± 0.0	0 ± 0.0	0 ± 0.0	0 ± 0.0	18 ± 6.0	0 ± 0.0	0 ± 0.0
		*abfB*	GH54	g3885.t1	42 ± 7.0	8 ± 3.3	7 ± 2.3	166 ± 23	125 ± 18	78 ± 13	723 ± 33	60 ± 7.3	20 ± 3.3
		*abfC*		g4219.t1	0 ± 0.0	0 ± 0.0	0 ± 0.0	0 ± 0.0	0 ± 0.0	1 ± 0.0	0 ± 0.0	0 ± 0.0	0 ± 0.0
	β-xylosidase: a-L-arabinofuranosidase	*abfD*	GH43	g4234.t1	4 ± 1.6	0 ± 0.0	8 ± 1.1	10 ± 2.7	22 ± 4.3	22 ± 3.8	2 ± 1.3	3 ± 2.0	16 ± 3.4
	a-glucuronidase	*aguA*	GH67	g5372.t1	10 ± 1.6	4 ± 1.7	19 ± 2.0	92 ± 8.4	94 ± 5.4	135 ± 10	14 ± 3.1	22 ± 2.8	21 ± 2.5
	Feruloyl esterase	*faeA*		g7596.t1	8 ± 2.1	0 ± 0.0	7 ± 1.8	4 ± 0.75	2 ± 0.8	4 ± 1.9	2 ± 1.1	3 ± 1.0	0 ± 0.0
		*faeB*		g10211.t1	6 ± 1.7	0 ± 0.0	10 ± 2.1	6 ± 2.3	6 ± 1.0	1 ± 0.5	5 ± 1.6	0 ± 0.0	9 ± 2.8
	Acetylxylan esterase	*axeA*		g7679.t1	3 ± 0.8	0 ± 0.0	4 ± 1.2	38 ± 6.6	141 ± 15	30 ± 9.0	5 ± 1.4	6 ± 1.1	15 ± 2.9

*PSM, Peptide Spectrum Matches; sd, standard deviation*.

On glycerol, glucose, and cellobiose, most xylan-degrading enzymes were expressed at basal levels, consistent with the low xylanase activity and weak xylanase bands (Figure 1 and Figure S2). Additionally, on glucose and cellobiose, the expression of two side-chain-degrading enzymes, AbnB (GH43; g3804.t1) and AxhA (GH62; g1234.t1), was induced at medium levels.

Based on enzyme-activity (Figure 1) and zymography (Figure S2) results, xylose, XOS, and xylan were good inducers of endoxylanase production as previously reported in *Trichoderma reesei, N. crassa*, and *Aspergillus nidulans* (Beg et al., 2001), but XOS and xylan were more effective than xylose at triggering the highly efficient expression of xylanases. Additionally, the efficient inducing capability of insoluble xylan might be attributed to the continuous release of XOS during the xylan-degradation process (Gong et al., 2016b), given the critical role played by the constant release of XOS from xylan. The XOS variants included substituted and unsubstituted species, with the short substituted XOS species potentially capable of being transported into cells as described in *Paenibacillus* spp. (Sawhney et al., 2014) to strongly induce the expression of side-chain-degrading enzymes, such as AxhA (GH62; g1234.t1) and AxeA (g7679.t1). The concentration of XOS-induced AxhA (GH62) was 2.5-fold higher than that induced by xylose and xylan; however, arabinose strongly induced the expression of AbfB from the GH54 family at concentrations 4- to 7-fold higher than that induced by xylose, XOS, and xylan. The different responses of AbfB and AxhA to carbon sources might reflect their discrepancies in substrate specificity as reported previously (van den Brink and de Vries, 2011).

Xylitol is a key common metabolite of xylose and arabinose; however, it only slightly induced the formation of xylan-degrading enzymes, suggesting that xylitol was converted to trace amounts of other compounds, such as xylose, arabinose, or L-arabitol, to function as inducers (Battaglia et al., 2011). In agreement with zymography results, CMC mainly induced the production of XynC (PSM = 87 ± 9.0) and XynB (PSM = 146 ± 15), but had no significant positive effect on the induction of XynA (PSM = 20 ± 4.0).

### Alterations in sugar-transporter expression based on time and carbon sources

**Figure 3:**
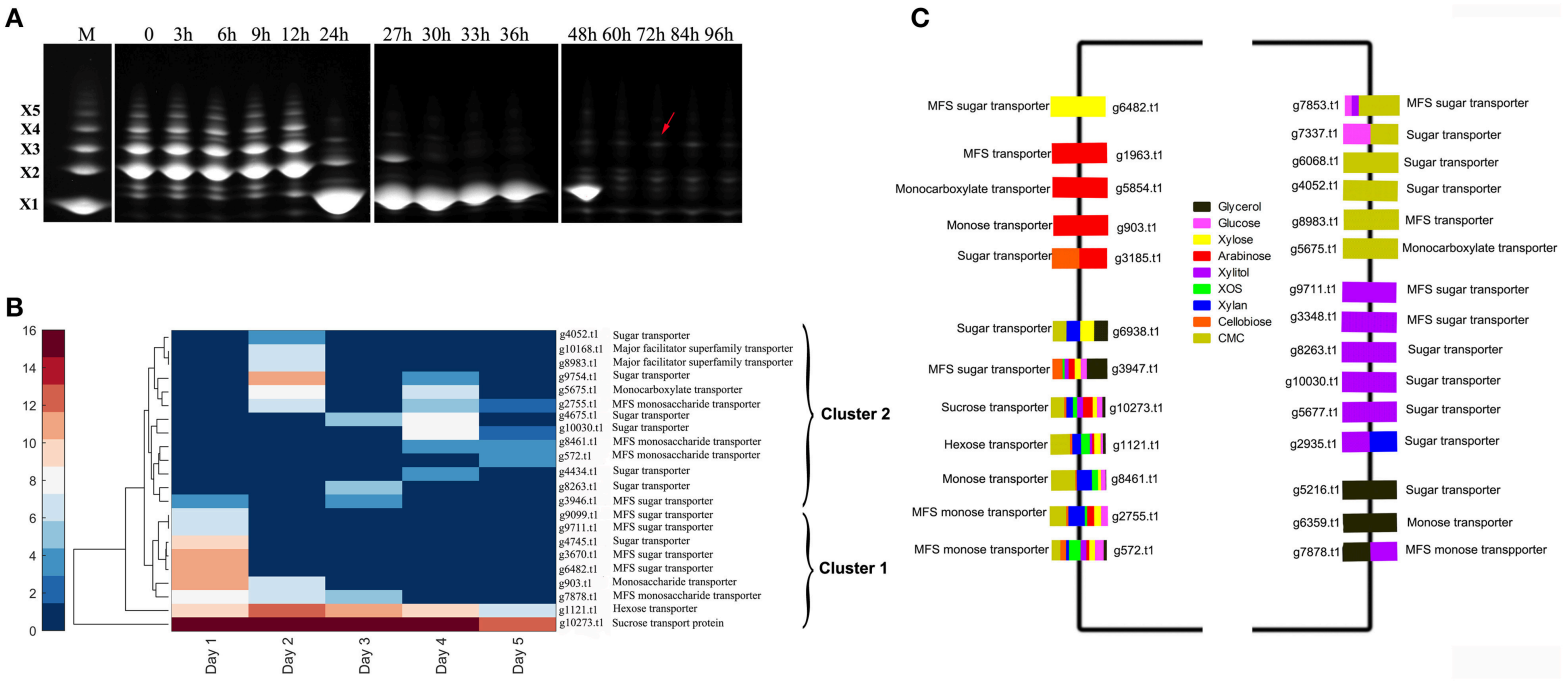
Detection of the change in extracellular reducing sugars and sugar transporters in *Aspergillus niger* An76 cultured on XOS and different carbon sources. **(A)** Time-course analysis of extracellular reducing sugars of *Aspergillus niger* An76 grown on 1% XOS detected by FACE. The splicing position between two FACE images is represented by white lines. **(B)** Sugar transporters identified over time in the presence of XOS detected by LC-MS/MS. **(C)** Sugar transporters detected on different carbon sources.

**Figure 4:**
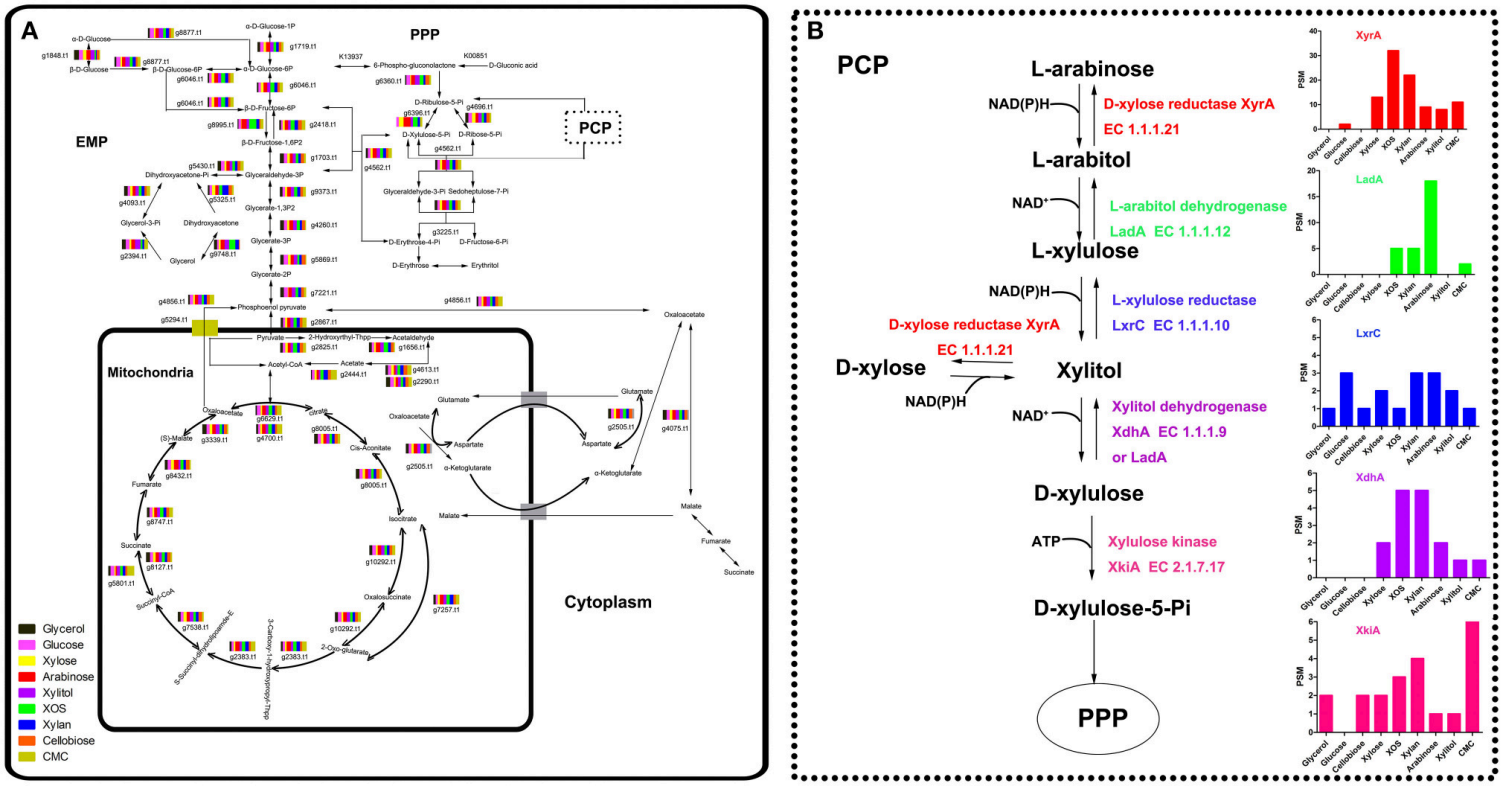
Intracellular enzymes involved in carbon metabolism in *Aspergillus niger* An76 cultivated on different carbon sources on day 5 detected with LC-MS/MS **(A)**. The dashed line represents the expression level of enzymes involved in the pentose catabolic pathway **(B)**.

The production of extracellular hydrolases is closely related to the concentration of imported sugars (Mach-Aigner et al., 2012). Analysis of the change in XOS species over time by FACE (**Figure 4A**) revealed that xylobiose, xylotriose, and xylotetraose were the main reducing sugars associated with the XOS substrate. From 12 to 24 h (1 day), the XOS with higher degree of polymerization were degraded, resulting in xylose accumulation, whereas in later stages, large amounts of substituted XOS were detected (Figure 3A, arrows; Gong et al., 2016b). Enzyme activity, zymography results, and MS data demonstrated that different enzymes were secreted in order, suggesting that the differences in expression of extracellular xylan-degrading enzymes were closely related to intracellular-transported sugars.

Predicted sugar transporters detected over time in the presence of XOS were clustered into two branches (clusters 1 and 2; Figure 3B, Table S5). In cluster 1, the annotated sucrose-transport protein (g10273.t1) and hexose transporter (g1121.t1) were continuously detected at high levels (PSM ≥ 10), suggesting that they were constitutively expressed and essential for allowing *A. niger* An76 to obtain readily available carbon sources. For some predicted transporters, such as annotated monosaccharide transporters (g903.t1 and g7878.t1) and sugar transporters (g6482.t1, g9099.t1, g9711.t1, g3670.t, and g4745.t1), expression levels were high on day 1, but then decreased, which was compatible with the rapid change observed in XOS on day 1 as detected by FACE, implying that the predicted sugar transporters were candidates for importing XOS products derived from the xylan backbone. These results suggested that transporters in cluster 2 expressed later may be responsible for the transport of substituted XOS detected during the later stages (Figure 3A).

We then grouped sugar transporters detected in the presence of specific carbon sources on day 5 (Figure 3C, Table S6). Seven predicted sugar transporters (g572.t1, g2755.t1, g8461.t1, g1121.t1, g10273.t1, g3947.t1, and g6938.t1), including the annotated hexose transporter (g1121.t1) and sucrose-transport protein (g10273.t1), were identified at high levels, indicating their low substrate specificity and fundamental roles in carbon metabolism. The high expression levels of hexose and sucrose transporters might explain the superiority of glucose and sucrose in citric acid fermentation by *A. niger* (Papagianni, 2007). The predicted monosaccharide transporter (g903.t1) and the predicted major facilitator superfamily (MFS) transporter (g1963.t1) were specific to arabinose, and the predicted MFS sugar transporter (g6482.t1) was exclusive to xylose, further verifying their transport specificity. Additionally, six new predicted transporters were identified in the presence of xylitol and CMC. The efficient transport of unnatural sugar alcohols and catabolites of substituted polymers revealed the potent uptake system in transporting heterogeneous substituted and unsubstituted xylan catabolites in *A. niger*.

### Induction of enzymes related to intracellular carbon metabolism by different carbon substrates

The imported sugars preferentially entered glycolysis (EMP), the citrate cycle (TCA), and the pentose phosphate pathway (PPP) and disassimilated to produce energy for microbial growth. Therefore, regardless of the type of sugar assimilated, a metabolic balance among EMP (*P* = 0.9282), TCA (*P* = 0.0949), and PPP (*P* = 0.6573) was reached, especially during the later stages of cultivation. The species and concentrations of enzymes involved in EMP, TCA, and PPP induced by different carbon sources were similar (Figure 4A; Tables S7–S11), consistent with a previous report (Lu et al., 2010).

Despite the similarity in enzyme expression involved in EMP, TCA, and PPP, the expression of reductases/dehydrogenases involved in PCP (Battaglia et al., 2011) showed significant discrepancies on different carbon sources (*P* = 0.0006, Figure 4B; Table S12). In the xylose-catabolism pathway of *A. niger*, the expression of key enzymes D-xylose reductase (XyrA; g219.t1) and xylitol dehydrogenase (XdhA; g3399.t1) for xylose utilization were mainly triggered by XOS. The expression levels on XOS were 2- to 3-fold higher than those observed on xylose. In the arabinose-metabolism pathway, L-arabitol dehydrogenase (LadA; g1617.t1) is the critical enzyme necessary for maintaining the balance of NAD^+^/NADH to facilitate the acceleration of arabinose metabolism (Hahn-Hägerdal et al., 2007). The significantly higher expression of LadA on arabinose indicated that arabinose played an important role in enhancing arabinose metabolism. Additionally, XOS and xylan were more effective as compared with xylose at inducing LadA expression. In *A. niger*, XlnR and AraR regulated xylose and arabinose metabolism (Battaglia et al., 2011). These results suggested that XOS might be more efficient than xylose at inducing the transcription of *xlnR* or *araR*.

**Figure 5:**
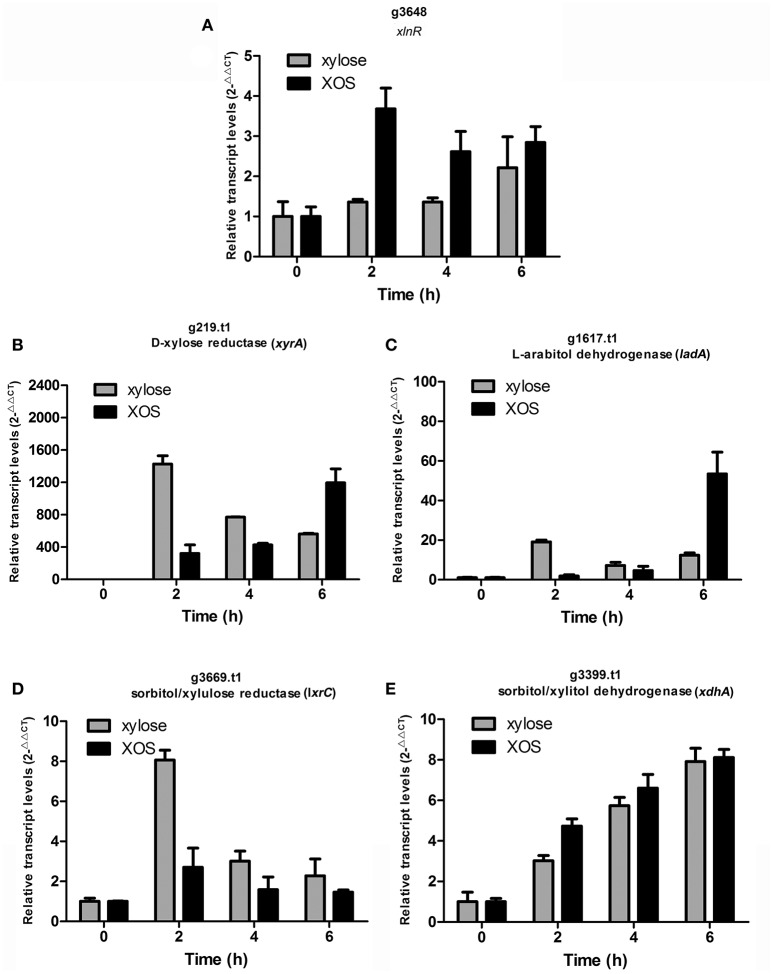
Time-course analysis of transcript levels of genes encoding **(A)** transcription activator (*xlnR*; g3648.t1), **(B)** D-xylose reductase (*xyrA*; g219.t1), **(C)** L-arabitol dehydrogenase (*ladA*; g1617.t1), **(D)** xylulose reductase (*lxrC*; g3669.t1), and **(E)** xylitol dehydrogenase (*xdhA;* g3399.t1) by qPCR in *Aspergillus niger* An76 induced using 1% xylose or 1% XOS. Glyceraldehyde-3-phosphate dehydrogenase (*gapdh*; g7576.t1) was used as reference gene. The results were calculated as the relative transcription level using the 2^−ΔΔCt^ method. Values represent the mean from two independent biological experiments measured in triplicate, and error bars indicate the standard deviations.

The differential effects of XOS and xylose on the transcription of the critical transcription activator x*lnR* were further explored by qPCR. Time-dependent transcription analysis revealed that the expression of x*lnR* induced by xylose was consistently low as previously reported (Mach-Aigner et al., 2012), whereas x*lnR* was expressed at relatively higher levels in the presence of XOS (*P* = 0.0314, Figure 5A). Proteome analysis also revealed that XlnR (g3648.t1) was detected when *A. niger* An76 was grown on XOS (Table S13), but absent on xylose. Additionally, the transcriptional discrepancies among *xyrA, xdhA, ladA*, and *lxrC* were analyzed over a time course (Figures 5B–E) at the transcriptional level. During the initial 2 h of culture, xylose was more effective at inducing the transcription of *xyrA, ladA*, and *lxrC* as compared with XOS; however, higher transcript levels were observed at 6 h in the presence of XOS. Based on the intracellular location of the enzymes involved in the PCP, they were more easily attacked by intracellular proteases; therefore, the delayed genetic expression regulated by XOS might result in the higher concentrations of enzymes associated with the PCP detected by LC-MS/MS on day 5.

## Discussion

**Figure 6:**
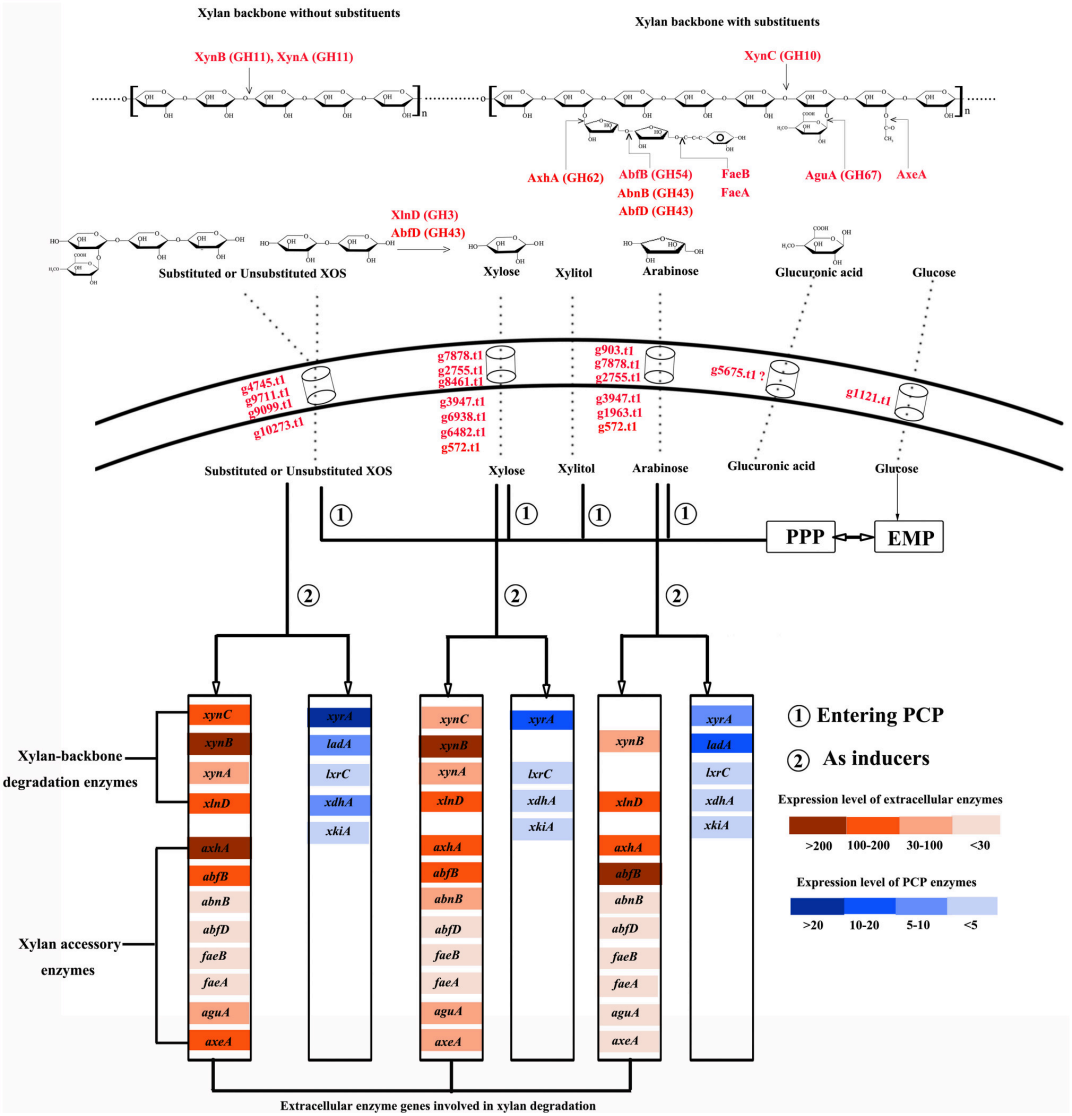
Schematic diagram of the xylan-degradation and -utilization strategies of *Aspergillus niger* An76.

In earlier studies, xylose was identified as inducing substance for the expression of xylanase and enzymes involved in xylose metabolism (Hasper et al., 2000; Mach-Aigner et al., 2012). Complex substrates, such as wheat straw and sugar cane bagasse, are more effective than monomeric sugars at inducing the transcription of xylan-degrading enzymes in *A. niger* (Salazar et al., 2009); however, it remains difficult to differentiate other key functional components. In this study, the products of complex xylan obtained during different disassembly periods were utilized as the sole carbon sources to culture *A. niger* An76. Based on our results, we showed that XOS was another critical molecule capable of activating the xylan-utilization system of *A. niger* An76 (Figure 6).

Due to the complex structure of xylan, microbes have co-evolved efficient and economical utilization systems (Sarkar et al., 2009). In *A. niger* An76, we detected the complete enzyme system required for xylan degradation and diverse isozymes with distinct physiological properties. Additionally, the expression order of backbone-degrading enzymes preceded that of side-chain-cleaving enzymes. XynC is induced by low xylose concentration (Xing et al., 2013); therefore, trace amounts of xylose in xylan-rich ambient triggered the secretion of XynC and released shorter substituted and unsubstituted XOS (Gong et al., 2016b). This was followed by the induced expression of XynA and XynB, with smaller molecular weights and higher efficiency on unsubstituted xylan backbones (Gong et al., 2016b), by imported XOS and their cooperative processing of the easily accessible unsubstituted backbone into diverse XOS species as described in induction models of *A. niger* (Delmas et al., 2012). Furthermore, the presence of XOS was responsible for the expression of β-D-xylosidase (XlnD), which acts synergistically with XynA and XynB to degrade unsubstituted and substituted XOS into xylose and shorter derivatives (Gong et al., 2016b). Therefore, based on the activities of backbone-degrading enzymes, xylan was cleaved into xylose and smaller XOS species.

Our previous study showed that substituted XOS was unable to be thoroughly degraded by XynA, XynB, XynC, and XlnD (Gong et al., 2016b). The release of smaller substituted XOS species indicated the presence of side chains and subsequently induced the expression of arabinose-releasing isozymes and glucuronidases. The expression of iso-arabinosidase varied on distinct substrates, indirectly reflecting its substrate specificity and roles in removing arabinose substitutes.

As previously reported (van den Brink and de Vries, 2011), AbfB (GH54-CBM42) and AxhA (GH62) were the main arabinose-degrading enzymes, with the expression of AxhA under the control of both XlnR and AraR, whereas only AraR is responsible for AbfB transcription (Battaglia et al., 2011), which may explain the higher expression levels of AxhA and AbfB induced by XOS and arabinose, respectively. Arabinofuranohydrolases in the GH62 family specifically cleave L-arabinose residues attached to arabinoxylan with α-1,2- or α-1,3-linkages, but are sensitive to adjacent substituents (van den Brink and de Vries, 2011); therefore, longer arabinan side chains and closely contiguous substituents were unable to be removed by AxhA. The rapid release of arabinose triggered the expression of AbfB, which immediately attacked arabinose. Due to the presence of CBM42, arabinose and arabinan linked to xylan were released (Vries and Visser, 2001); therefore, the arabinan-substituted XOS could be further acted upon by AbfB. 4-*O*-methyl-glucuronic acid is another important xylan substituent capable of being removed by glucuronidases, whereas glucuronidase was reported to be only able to degrade the substituted XOS rather than xylan (van den Brink and de Vries, 2011). This was consistent with results showing that the expression of glucuronidases occurred after that of the backbone-degrading enzymes.

The pentose sugars (xylose and arabinose) or XOS released by extracellular enzymes from xylan induced the distinct expression of sugar transporters as reported in *T. reesei* (Gupta et al., 2016). Sugar transporters play a central role in importing heterogeneous hydrolysates and controlling the expression of xylan-degradation enzymes in response to xylan (Gupta et al., 2016). The time-dependent expression discrepancies observed in the sugar transporters in the presence of XOS were consistent with the different secretion order of the enzymes. The transporters expressed during the initial 2 days in the presence of XOS may be primarily responsible for the uptake of unsubstituted XOS and xylose, whereas those detected during the later stages might be related to the transport of substituents and substituted XOS. This might explain the abundant transporters detected in the presence of CMC and xylitol on day 5. In this study, we identified several predicated hexose, pentose, or XOS transporters, which constituted good candidates as sugar transporters for engineering pentose fermentation in *Saccharomyces cerevisiae*.

As shown in Figure 6, most of the imported XOS, xylose, and arabinose species entered the PCP, PPP, and EMP, whereas only traces or their metabolic intermediates acted as transcriptional inducers of the enzymes involved in xylan degradation and metabolism. Compared with xylose and arabinose, XOS induced expression of additional xylan-backbone- and side-chain-degrading enzyme genes as well as enzymes participating in the PCP. It was confirmed that the transcriptional activator XlnR regulates the expression of both xylan-degrading enzymes and enzymes involved in the PCP in *A. niger* (Hasper et al., 2000), but that XlnR-induced expression in the presence of xylose of *A. niger* xylanase genes was also modulated by CreA; therefore, the higher expression level of these genes on xylan than that on xylose was usually attributed to the repression effects of high xylose concentrations as explained previously (Vries et al., 1999). However, analysis of the effects of xylose concentration on the expression of xylan-backbone-degrading enzymes secreted by *A. niger* An76 revealed that a high xylose concentration was favorable for certain groups of genes (*xynA* and *xynB*) (Xing et al., 2013), agreeing with results reported by Mach-Aigner et al. (2012). Therefore, the higher expression levels of *xynA* and *xynB* induced by XOS may not entirely depend upon the release of low concentrations of xylose, but rather result from the higher transcription levels of XlnR triggered by imported XOS, as shown from transcription and proteome analyses.

In *A. niger*, XlnR exists in an inactive state in the cytoplasm under non-inducing conditions, but is imported into the nucleus upon induction by xylose (Hasper et al., 2004). Additionally, in *T. reesei, de novo* biosynthesis of Xyr1 and its simultaneous nuclear import are required for xylanase gene expression (Lichius, 2014). Therefore, the high expression levels of XlnR induced by XOS might be related to the higher efficiency of interactions observed between intracellular XOS and inactive XlnR as compared with those involving xylose. These interactions activated XlnR and initiated its entry into the nucleus to trigger the *de novo* biosynthesis of XlnR.

A previous study showed that XlnR was capable of regulating the expression of *xyrA* and extracellular xylan-degrading enzymes in *A. niger* (Hasper et al., 2000). Moreover, XlnR interactions with the *xyrA* promoter lead to decreased XlnR-induced expression of extracellular xylan-degrading enzymes (Hasper et al., 2000). The discrepancies in time-dependent expression of genes encoding enzymes associated with the PCP involved enzymes induced by XOS and xylose and suggested that during the initial stages, small concentrations of XlnR induced by xylose may primarily target the promoters of *xyrA, ladA*, and *lxrC*. By contrast, our findings showed that XOS induced relatively higher levels of XlnR expression, despite small concentrations capable of inducing the expression of enzymes associated with the PCP; therefore, during the initial stage, XOS was more effective as compared with xylose at inducing XynA and XynB expression (Figures S2B,E). Overall, the higher efficiency of XOS at activating the xylanolytic system of *A. niger* An76 might result from the efficient induction of XlnR and the functions associated with substituted XOS involved in improving the species-specific expression and concentrations of xylan side-chain-degrading enzymes.

In other filamentous fungi, such as *T. reesei* and *Penicillium oxalicum*, large amounts of oligosaccharides were detected during the initial stages when cultured on corn stover and wheat bran, resulting in the identification of diverse isozymes exhibiting different structures and secretion orders (Gong et al., 2015). Therefore, similar xylan-degradation strategies as those illustrated in *A. niger* An76 might be present in other filamentous fungi. Based on the highly efficient xylan-utilization strategy observed in *A. niger* An76, it might be possible to improve the species-specific expression and concentrations of xylan-degrading enzymes and shorten fermentation time by adding abundant intermediate products of xylan (XOS) to cultures of filamentous fungi; however, the mechanisms associated with how XOS functions and which kind of XOS (xylobiose, xylotriose, xylotetraose, xylopentaose, substituted XOS) playing a dominant inducing role requires further study, in addition, to completely exclude the effect of low quantities of xylose on XOS direct induction of XlnR, mutants with beta-xylosidase, arabinose-releasing enzymes and α-glucuronidase knocked out would be very valuable in this regard, what's more, in *N. crassa*, it has indicated that *xlr*-1 is regulated by a combination of induction and derepression and that *xlr*-1 is also subject to non-CRE-1-mediated carbon catabolite repression (CCR) (Sun et al., 2012), therefore, the effects of CCR on the expression of *xlnR* deserve further exploration.

Here, we comprehensively studied the synergistic and coordinated expression of xylan-degrading enzymes, sugar transporters, and intracellular enzymes related to xylan metabolism. Our results will fundamentally influence the understanding of efficient xylan-degradation and -utilization mechanisms in filamentous fungi. Furthermore, these findings revealed how complex substrate induce the expression of degradative enzymes in fungi and will aid in mining of xylan backbone degradation enzymes and side-chain releasing enzymes, the complete side-chain releasing enzymes secreted by *A. niger* An76 will improve the efficiency of substituents degradation, and the enzymes were expected to be engineered in some fungi such as yeast by metabolic engineering to convert xylan into biofuels and biochemicals directly.

## Author contributions

LW designed the experiments. WG and LD performed the experiments. WG, HZ, LZ, and LW analyzed the data and wrote the manuscript. All authors read and approved the final manuscript.

### Conflict of interest statement

The authors declare that the research was conducted in the absence of any commercial or financial relationships that could be construed as a potential conflict of interest.

## Abbreviations

XOS: xylooligosaccharide
PCP: pentose catabolic pathway
CMC: carboxymethylcellulose sodium
FACE: fluorescence-assisted carbohydrate electrophoresis
PAGE: polyacrylamide gel electrophoresis
LC-MS/MS: liquid chromatography tandem mass spectrometry
qPCR: quantitative polymerase chain reaction
PSMs: peptide spectrum matches
MFS: major facilitator superfamily.
