# Supplementary material for: A Highly Efficient Xylan-Utilization System in Aspergillus niger An76: A Functional-Proteomics Study
Source: Front Microbiol. 2018 Mar 22;9:430. doi: 10.3389/fmicb.2018.00430 (PMC5874446; doi:10.3389/fmicb.2018.00430)
Supplement: Supplementary file 1 [file Table1.DOCX]

**Table S1.** Forward and reverse primers used for qPCR.

| Genome ID | Gene name | Activity | Primer sequences |
| --- | --- | --- | --- |
| g7576.t1 (reference gene) | *gapdh* | Glyceraldehyde-3-phosphate dehydrogenase | F: ATTTTGGTGTTGCTCAGGG |
|  |  |  | R: CGGCGGTTCTTCTTGCTAT |
| g3648.t1 (target gene) | *xlnR* | Transcription activator | F: CCCGACGAAACCCCGAATA |
|  |  |  | R: GGGCGGCGATGTCAAGAAA |
| g219.t1(target gene) | *xyrA* | D-xylose reductase | F: ATCCCTACCTCACGCAGAC |
|  |  |  | R: ACTCAACGGTCCAAAAGAA |
| g1617.t1(target gene) | *ladA* | L-arabitol dehydrogenase | F: ATGTCACCTCCCTCAAGCC |
|  |  |  | R: CCGTTGTAGCGACCAGTCA |
| g3399.t1(target gene) | *xdhA* | Sorbitol/Xylitol dehydrogenase | F: TATTGGGCTGCTGTCGTCG |
|  |  |  | R: AATGCCTCTTCCGCCTGTG |
| g3669.t1(target gene) | *lxrC* | Sorbitol/Xylulose reductase | F: GGCAAGGTCGTCGTTATTA |
|  |  |  | R: CGAGAAGAGTAGGTGAGGG |
