# Supplementary material for: A Highly Efficient Xylan-Utilization System in Aspergillus niger An76: A Functional-Proteomics Study
Source: Front Microbiol. 2018 Mar 22;9:430. doi: 10.3389/fmicb.2018.00430 (PMC5874446; doi:10.3389/fmicb.2018.00430)
Supplement: Supplementary file 14 [file Image1.PDF]

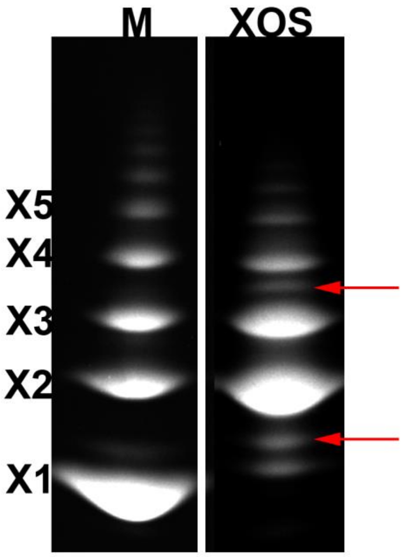

Figure S1. Detection of the composition of XOS by FACE

M: standard substance; X1, X2, X3, X4, X5: xylose, xylobiose, xylotriose, xylotetraose, xylopentaose; the white line indicated the splicing position between two FACE images
