# Supplementary material for: A Highly Efficient Xylan-Utilization System in Aspergillus niger An76: A Functional-Proteomics Study
Source: Front Microbiol. 2018 Mar 22;9:430. doi: 10.3389/fmicb.2018.00430 (PMC5874446; doi:10.3389/fmicb.2018.00430)
Supplement: Supplementary file 15 [file Image2.PDF]

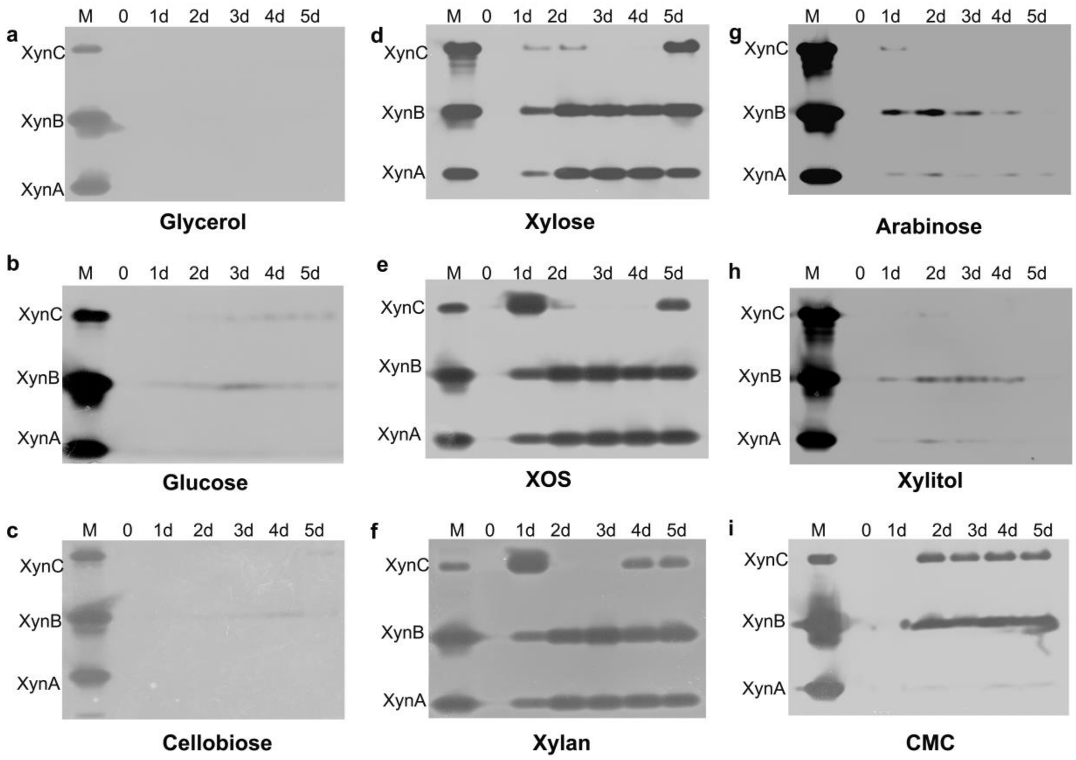

Figure S2. Native PAGE displaying the dynamic zymography results of xylanases secreted by *Aspergillus niger* An76 grown on the following listed carbon sources: (a) glycerol, (b) glucose, (c) cellobiose, (d) xylose, (e) XOS, (f) xylan, (g) arabinose, (h) xylitol, and (i) CMC
