# Supplementary material for: A Highly Efficient Xylan-Utilization System in Aspergillus niger An76: A Functional-Proteomics Study
Source: Front Microbiol. 2018 Mar 22;9:430. doi: 10.3389/fmicb.2018.00430 (PMC5874446; doi:10.3389/fmicb.2018.00430)
Supplement: Supplementary file 16 [file Image3.PDF]

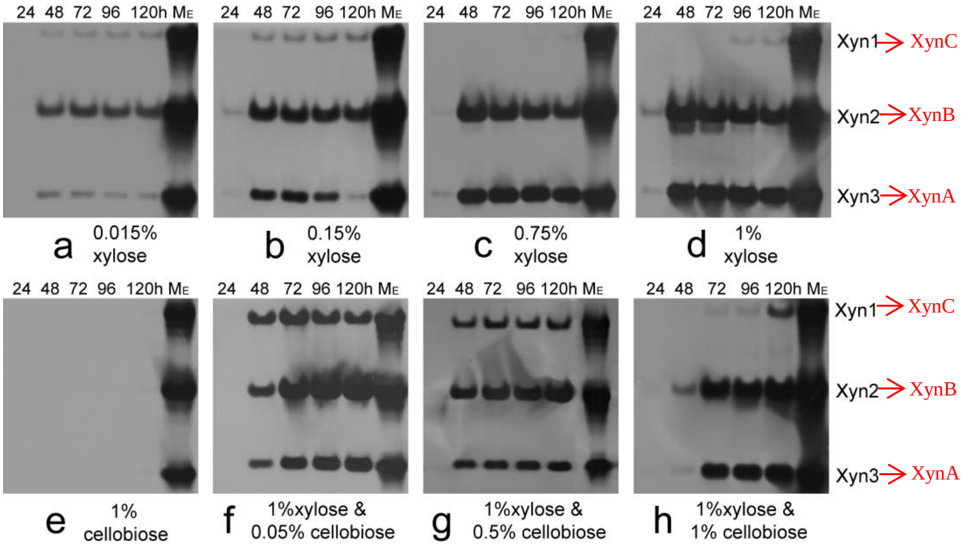

Figure S3. The history of renaming of the three xylanases separated from *A. niger* An76 and detected by native-PAGE (Xing S, Li G, Sun X, et al. Applied Biochemistry & Biotechnology, 2013, 171(4):832-846); The three xylanases were named after Xyn1, Xyn2 and Xyn3 in previous study (black), and they were renamed as XynC, XynB and XynA , respectively, in this study (red).
