# Supplementary material for: A Highly Efficient Xylan-Utilization System in Aspergillus niger An76: A Functional-Proteomics Study
Source: Front Microbiol. 2018 Mar 22;9:430. doi: 10.3389/fmicb.2018.00430 (PMC5874446; doi:10.3389/fmicb.2018.00430)
Supplement: Supplementary file 17 [file Image4.PDF]

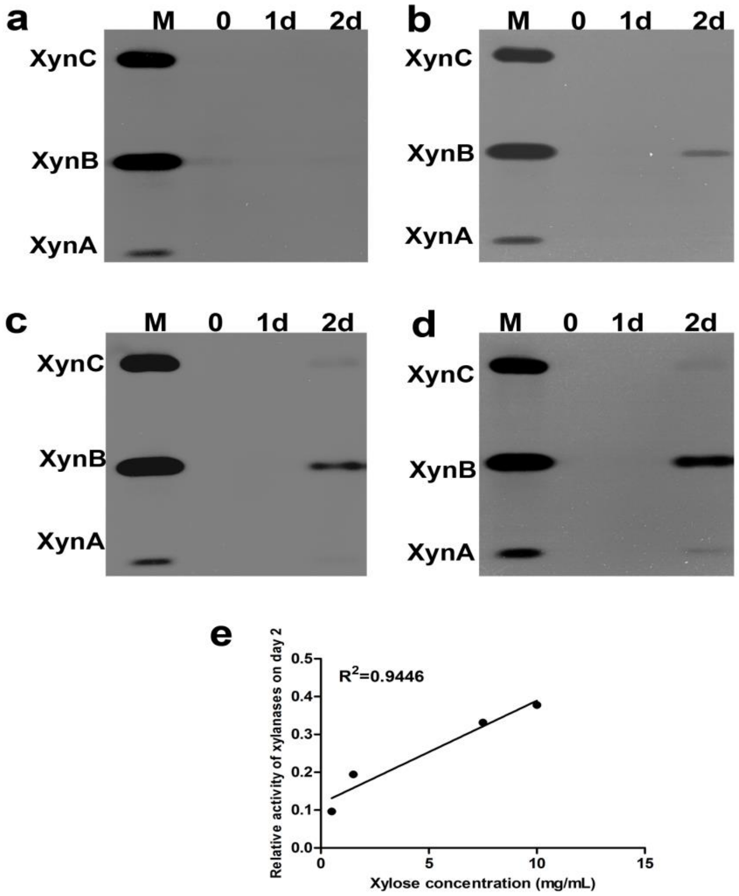

Figure S4 Xylanases produced in the initial 2 days detected with native-PAGE. (a) 0.05% xylose; (b) 0.15% xylose; (c) 0.75% xylose; (d) 1% xylose; (e) Correlation analysis of the relative xylanase activity on day 2 with xylose concentration.
