# Supplementary material for: A Highly Efficient Xylan-Utilization System in Aspergillus niger An76: A Functional-Proteomics Study
Source: Front Microbiol. 2018 Mar 22;9:430. doi: 10.3389/fmicb.2018.00430 (PMC5874446; doi:10.3389/fmicb.2018.00430)
Supplement: Supplementary file 18 [file Image5.PDF]

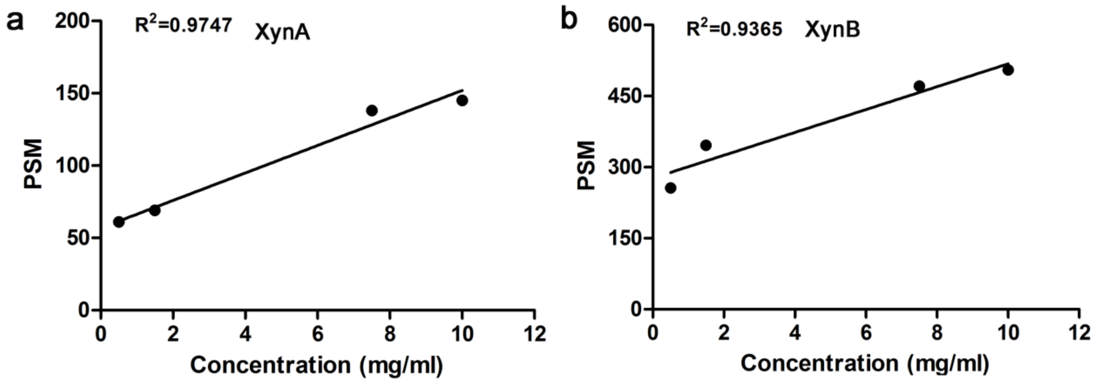

Figure S5. Correlation analysis of the expression level of (a) XynA (a) and (b) XynB with xylose concentration. PSM: Peptide Spectrum Matches
